# Protective Roles for Potassium SK/K_Ca_2 Channels in Microglia and Neurons

**DOI:** 10.3389/fphar.2012.00196

**Published:** 2012-11-26

**Authors:** Amalia M. Dolga, Carsten Culmsee

**Affiliations:** ^1^Institute of Pharmacology and Clinical Pharmacy, University of MarburgMarburg, Germany

**Keywords:** calcium regulation, K_Ca_2/SK channels, K_Ca_3/IK channels, microglia, neuroprotection

## Abstract

New concepts on potassium channel function in neuroinflammation suggest that they regulate mechanisms of microglial activation, including intracellular calcium homeostasis, morphological alterations, pro-inflammatory cytokine release, antigen presentation, and phagocytosis. Although little is known about voltage independent potassium channels in microglia, special attention emerges on small (SK/*KCNN1*-3/K_Ca_2) and intermediate (IK/*KCNN4*/K_Ca_3.1)-conductance calcium-activated potassium channels as regulators of microglial activation in the field of research on neuroinflammation and neurodegeneration. In particular, recent findings suggested that SK/K_Ca_2 channels, by regulating calcium homeostasis, may elicit a dual mechanism of action with protective properties in neurons and inhibition of inflammatory responses in microglia. Thus, modulating SK/K_Ca_2 channels and calcium signaling may provide novel therapeutic strategies in neurological disorders, where neuronal cell death and inflammatory responses concomitantly contribute to disease progression. Here, we review the particular role of SK/K_Ca_2 channels for [Ca^2+^]_i_ regulation in microglia and neurons, and we discuss the potential impact for further experimental approaches addressing novel therapeutic strategies in neurological diseases, where neuronal cell death and neuroinflammatory processes are prominent.

## Introduction

An increasing number of recent reports on potassium SK/K_Ca_2 and IK/K_Ca_3.1 channels elucidate their multifaceted functions in cell motility, neuronal excitability, neuroprotection, and neuroinflammation (Stocker, 2004; Schlichter et al., 2010; Allen et al., 2011; Dolga et al., 2011). In fact, based on the plethora of functions exerted by SK/K_Ca_2 channels, modulators of these potassium channels have been proposed for therapeutic applications in a wide variety of pathological conditions, including brain tumors, cerebral ischemia, schizophrenia, Parkinson’s and Alzheimer’s diseases, and other neurological diseases where neuroinflammatory mechanisms are considered as major hallmarks (see Table 1; Rupalla et al., 1998; Wyss-Coray and Mucke, 2000; Liégeois et al., 2003; Yan et al., 2003; Stocker, 2004; Judge et al., 2007; Hirsch and Hunot, 2009; Schlichter et al., 2010; Allen et al., 2011; Dolga et al., 2011, 2012; Varas-Lorenzo et al., 2011; Herrik et al., 2012; Kuiper et al., 2012). For example, riluzole, a drug used in patients with amyotrophic lateral sclerosis (ALS) and hereditary cerebellar ataxia, is a potent activator of SK/K_Ca_2 channels (Grunnet et al., 2001; Cao et al., 2002) that reduces neuroinflammation and neuronal excitability (Judge et al., 2007; Waubant, 2007; Ristori et al., 2010; Liu et al., 2012; see Table 1). Recently, derivatives of 2-(phenylamino)benzimidazole and 2-amino benzimidazole were patented as novel SK/K_Ca_2 channel modulators for therapeutic applications in Alzheimer’s disease, anxiety, and ataxia (Sørensen et al., 2006a,b).

**Table 1:** **Targeting K_Ca_2.1/K_Ca_2.2/K_Ca_2.3 channels in CNS disorders – patents and clinical trials**.

Type of action	Compound	Diseases/pathological conditions	Reference
Activation	Riluzole	Hereditary ataxia, multiple sclerosis, cerebellar ataxia	Grunnet et al. (2001), Cao et al. (2002), Ristori et al. (2010), Waubant (2007)
Inhibition	2-(Phenylamino) benzimidazole, 2-amino benzimidazole derivatives	Cerebral ischemia, stroke, AD, ataxia, anxiety, depression, cognitive disorders, hearing loss	Sørensen et al. (2006a,b), Wang et al. (2003)
Inhibition	Pyrazolyl-pyrimidine derivatives	Epilepsy, convulsions neuroinflammation	Eriksen et al. (2006)

## Potassium Channels in Microglia

Patch-clamp studies of microglial cells showed that these cells express a wide variety of potassium channels. These include inward rectifier K^+^ (Kir) channels (described in rat, murine, bovine, and human microglia), delayed outwardly rectifying K^+^ (Kdr) channels (described in rat, mouse, and human microglia), human ether-a-go-go-related gene (HERG) K^+^ channels (described in rat microglia), G protein-activated K^+^ channels, ATP-sensitive potassium (K_ATP_) channels, and voltage independent calcium-activated potassium (SK/K_Ca_2) channels (described in murine, bovine, and human microglia; Eder et al., 1997; Eder, 1998). Several studies demonstrated that microglial potassium channel activity promotes neuronal survival and reduces microglial activation and related neuroinflammation (Polazzi and Monti, 2010; Kettenmann et al., 2011). For example, K_ATP_ channel activation using diazoxide exerted anti-inflammatory effects in LPS and interferon gamma (IFNγ)-activated mouse primary microglial cells *in vitro* (Virgili et al., 2011). Diazoxide, a classical K_ATP_ channel activator, prevented rotenone-induced rat microglial activation (Zhou et al., 2008), inhibited NO release, tumor necrosis factor alpha (TNF-α), interleukin-6 (IL-6) production, and inducible nitric oxide synthase (iNOS) expression in a mouse model for multiple sclerosis in an induced experimental autoimmune encephalomyelitis (Virgili et al., 2011).

### KCNN3/SK3/K_ca_2.3 channel function

The expression of all subtypes *KCNN1*-3/SK1-3/K_Ca_2.1–3 channels have been described in microglial cells (Kaushal et al., 2007; Schlichter et al., 2010; Hayashi et al., 2011). However, among all SK1-3/K_Ca_2.1–3 channel subtypes, the SK3/K_Ca_2.3 channel subtype plays the most critical role in microglial activation. The mRNA of SK3/K_Ca_2.3 channels was detected in mouse and rat microglial cells (Khanna et al., 2001; Dolga et al., 2012) and was found significantly up-regulated after LPS stimulation in rat primary microglial cells (Schlichter et al., 2010). In recent studies, contributions of SK3/K_Ca_2.3 channels to microglial activation processes were elucidated using a pharmacological approach (Schlichter et al., 2010) based on affinities of apamin and tamapin for cloned SK/K_Ca_2 channels (Pedarzani and Stocker, 2008). In these experiments, 100 nM apamin or 5 nM tamapin inhibited the function of SK2/K_Ca_2.2 and SK3/K_Ca_2.3 channels, while the role for SK3/K_Ca_2.3 channels was deduced when the cellular microglial functions were compared with tamapin at concentrations of 250 pM that should not inhibit SK3/K_Ca_2.3 channel function (Schlichter et al., 2010). Using this pharmacological approach, the authors found that inhibition of SK3/K_Ca_2.3 channels reduced the neurotoxic effects of activated rat primary microglia.

In the same study, apamin or tamapin reduced LPS-induced NO release, and decreased the activity of p38-mitogen-activated protein kinase (MAPK) in microglia cells and attenuated tyrosine-nitrated proteins in the neurons exposed to activated microglia. Interestingly, LPS-mediated nuclear factor-kappa B (NF-κB) activation was not affected by low doses of either apamin or tamapin (Schlichter et al., 2010).

Furthermore, it has not yet been determined how SK3/K_Ca_2.3 channel modulation regulates microglial activation pathways; including cytokine release and calcium deregulation and if there are any species-dependent differences. Since the previous studies using small molecules or toxins seem to reveal critical roles of SK3/K_Ca_2.3 channels in microglial activation, studies using SK/K_Ca_2 channel siRNA or knockout animals are required to resolve the limitations of these pharmacological approaches.

## Potassium KCNN/SK/K_ca_2 Channels in Neurons

All subtypes of small-conductance calcium-activated potassium *KCNN1*-3/SK1-3/K_Ca_2.1–3 channels have been identified in neuronal cells of different species, including mouse, rat, and human neurons (Köhler et al., 1996; Sailer et al., 2004; Dolga et al., 2011). In neuronal cells, SK/K_Ca_2 channels are activated by submicromolar calcium concentrations (*K*_D_ ∼ 0.5 μM) due to the calcium sensing binding site, a calmodulin (CaM)-binding domain located at the C-terminus of β-subunits (Adelman et al., 2012).

Functional SK/K_Ca_2 channels consist of four subunits, each with six transmembrane segments and cytosolic N and C termini (Stocker, 2004). Upon increasing intracellular calcium concentrations, CaM interaction to CaM-binding domain (CaMBD) alters the geometry of the C-lobe E-F hands from one subunit of SK/K_Ca_2 channels by binding to a neighboring subunit and forming a dimer that promotes a conformational rotation of CaMBD, allowing potassium flow through the channel pore (Xia et al., 1998; Adelman et al., 2012). It is well established that SK/K_Ca_2 channels mediate afterhyperpolarization (AHP) in response to an action potential in excitable cells. Opening of SK/K_Ca_2 channels promotes a negative resting membrane potential or even hyperpolarizing, preventing the onset of a second action potential. Thus, SK/K_Ca_2 channels act as fine tuning regulators of action potential frequencies, neuronal excitability, and [Ca^2+^]_i_ (Stocker, 2004).

SK/K_Ca_2 channels are responsible for the medium AHP (mAHP), which decays in 200 ms (Bond et al., 2004), while BK channels are responsible for the fast AHP component, which decays in 50 ms (Faber and Sah, 2003). SK/K_Ca_2 channels possess a small unitary conductance (∼10 pS in symmetrical potassium; Köhler et al., 1996) and exhibit an inward rectification. The general knowledge of the inward rectification of SK/K_Ca_2 channels is attributed to the block of outward current by divalent ions, such as Ca^2+^ and Mg^2+^ (Lu, 2004), as it is the case for inward rectifier K^+^ channels (Kir, IRK). However, it was recently shown by inside-out patch-clamp recordings of rat SK2/K_Ca_2.2 channel activity that three negatively charged residues in the sixth transmembrane domain could also be responsible for the SK/K_Ca_2 channel inward rectification (Li and Aldrich, 2011). Importantly, these negatively charged residues affect the Ca^2+^ affinity for SK/K_Ca_2 channel gating, and the open probability (*P*_o_) of the channel in the absence of Ca^2+^ and this intrinsic inward rectification is largely attributed to a voltage-dependent reduction in outward single-channel conductance (Li and Aldrich, 2011). However, in specific neurons, such as in the rat nucleus basalis, in the rat ventral midbrain, or in preoptic rat hypothalamus, SK/K_Ca_2 channels are responsible for slow “spontaneous miniature” outward currents (Arima et al., 2001; Cui et al., 2004; Klement et al., 2010). The precise function of these outward currents has not yet been elucidated, however, it has been suggested that they may also contribute to spontaneous firing, hyperpolarization, and regulation of burst firing (Adelman et al., 2012).

### SK2/K_ca_2.2 channel function

In hippocampal and cortical mouse neurons, SK2/K_Ca_2.2 channel subtypes are localized at dendritic spines, where they are closely associated with NMDA receptors and contribute to AHP, regulate calcium homeostasis and reduce the amplitude of evoked synaptic potential in an NMDA receptor-dependent manner, thereby attenuating neuronal excitability (Faber et al., 2005; Ngo-Anh et al., 2005). Very little has been reported on the regulation of SK2/K_Ca_2.2 channel expression under physiological conditions and in disease. The physiological regulatory functions of SK2/K_Ca_2.2 receptors on NMDAR function and calcium homeostasis become even more relevant under conditions of excitotoxic stress. For example, pharmacological activation of SK2/K_Ca_2.2 receptors attenuates NMDA-mediated intracellular [Ca^2+^] increases and excitotoxicity in rat primary cortical neurons (Dolga et al., 2011). In contrast, NMDAR-mediated excitotoxicity and cell death is associated with rapid SK2/K_Ca_2.2 down-regulation in both rat and mouse primary neurons (Allen et al., 2011; Dolga et al., 2011), and such reduced SK2/K_Ca_2.2 protein levels resulted in a failure to regulate neuronal excitability. Overall these data point at a pivotal regulative function of SK2/K_Ca_2.2 channels on NMDAR-dependent calcium homeostasis under physiological conditions and suggest a therapeutic potential in excitotoxic conditions.

Interestingly, two responsive elements of NF-κB were found in the intronic promoter region of SK2/K_Ca_2.2 channels, suggesting that NF-κB regulates SK2/K_Ca_2.2 channel gene transcription in rat adrenal medulla PC12 cells (Kye et al., 2007). In activated microglia, NF-κB signaling and the p38 MAPK pathway are strongly activated (Koistinaho and Koistinaho, 2002; Mattson, 2005). In addition, TNF-α production is also augmented following microglial activation (Kettenmann et al., 2011). In mouse primary cortical neurons, TNF-α facilitates activation of NF-κB signaling, which in turn may up-regulate SK2/K_Ca_2.2 channel gene transcription in neurons (Dolga et al., 2008). For example, it is not yet known whether NF-κB activity also modulates SK2/K_Ca_2.2 channel gene transcription in microglial cells. However, in activated rat primary microglial cells, inhibition of SK/K_Ca_2 channels by apamin further decreased inhibitory factor κB (IκB-α) levels (Schlichter et al., 2010). These data suggested that inhibition of SK/K_Ca_2 channels does not affect LPS-induced NF-κB activation in activated rat primary microglial cells. It is not yet resolved whether SK2/K_Ca_2.2 channel function is different in various mammalian species or whether in activated microglia, the persistent increase in [Ca^2+^]_i_ and TNF-α production is associated with altered SK2/K_Ca_2.2 channel expression and activity.

## Neuronal and Microglial Pathways of SK1-3/K_ca_2.1–3 Channel Activation

### Neuronal pathways

In excitable cells, SK/K_Ca_2 channels are activated by increases in intracellular calcium after release from several different sources: Ca^2+^ influx from the extracellular space via ionotropic receptors, including NMDARs and nicotinic acetylcholine receptors (nAChRs), Ca^2+^ influx via voltage-dependent calcium channels (VDCC, Cav), and Ca^2+^ release from the intracellular calcium stores, such as the endoplasmic reticulum (ER; Oliver et al., 2000; Ngo-Anh et al., 2005). For example, at the dendritic spines of mouse hippocampal neurons, NMDARs are closely associated with SK2/K_Ca_2.2 channels (Ngo-Anh et al., 2005). NMDARs-dependent Ca^2+^ transients induce excitatory postsynaptic currents that are followed by SK/K_Ca_2 channel activity in the rat brain (Faber et al., 2005). While the slow kinetics of the NMDARs does not provide the major Ca^2+^ source for SK/K_Ca_2 channel activation in rat hippocampal brain slices (Sabatini et al., 2002), Ca^2+^ influx through α9-nAChRs is direct and more effective in activating SK/K_Ca_2 channels in the rat basolateral amygdala (Power and Sah, 2008). In the inner and outer hair cells of the rat cochlea, SK/K_Ca_2 channels and nAChRs are functionally coupled and are situated ∼10–20 nm apart (Oliver et al., 2000). SK/K_Ca_2 channels translate Ca^2+^ influx through nAChRs inducing an inhibitory postsynaptic current that hyperpolarizes the plasma membrane and reduces action potential frequencies (Oliver et al., 2000). Action potentials induce Ca^2+^ influx via VDCC generating fast, large (larger than micromolar concentrations), and local Ca^2+^ increases. Activation of SK/K_Ca_2 channels through VDCC channels is largely attributed to their proximity to these channels (Fakler and Adelman, 2008).

Further, inositol 1,4,5-triphosphate (IP_3_) generation via metabotropic receptor activation is another neuronal Ca^2+^ source for SK/K_Ca_2 channel activation. IP_3_ generation in the presence of an action potential further promote Ca^2+^ release from the intracellular stores, such as ER, that in turn activates SK/K_Ca_2 channels (Power and Sah, 2008). Using whole-cell patch-clamp recordings and high-speed fluorescence imaging it was shown that muscarinic acetylcholine receptors (mAChR) agonists promote large rises in cytosolic calcium via IP3 receptors in the soma and proximal dendrites of neurons in the rat basolateral amygdala. This calcium release was associated with activation of an outward current and hyperpolarization, which resulted from the activation of SK/K_Ca_2 channel (Power and Sah, 2008). The functional relevance of this SK/K_Ca_2 channel activation was demonstrated in rat midbrain dopaminergic neurons, in rat cortical neurons, and rat amygdala, where glutamate and acetylcholine release in response to an action potential mediated a slow inhibitory postsynaptic potential, largely attributed to SK/K_Ca_2 channel activation via calcium release from the IP_3_ intracellular stores (Fiorillo and Williams, 1998; Morikawa et al., 2000; Seutin et al., 2000; Faber et al., 2005; Gulledge and Stuart, 2005).

Another calcium source for SK/K_Ca_2 channel activation is promoted by P/Q-type Ca^2+^ channel activity and ryanodine receptor (RyR)-induced Ca^2+^ release (Adelman et al., 2012). Mutations in P/Q-type Ca^2+^ channels are associated with human episodic ataxia type 2, irregular pacemaking, and reduced firing precision in mouse Purkinje cells. Reduced activity of P/Q-type Ca^2+^ channels is attributed to a decreased mAHP as a consequence of reduced activity of postsynaptic P/Q-coupled SK/K_Ca_2 channels. Indeed, *in vivo* application of the SK/K_Ca_2 channel opener 1-EBIO into the cerebellum significantly improved the motor coordination deficits, dyskinesia, and ataxia in the P/Q-type Ca^2+^ channel mutant mice (Walter et al., 2006). This particular cross-talk between SK/K_Ca_2 channels and P/Q-type Ca^2+^ channels is of critical importance in the cerebellum where together with RyR they cooperate to generate and maintain AHP (Kakizawa et al., 2007).

### Microglial pathways

**Figure 1:**
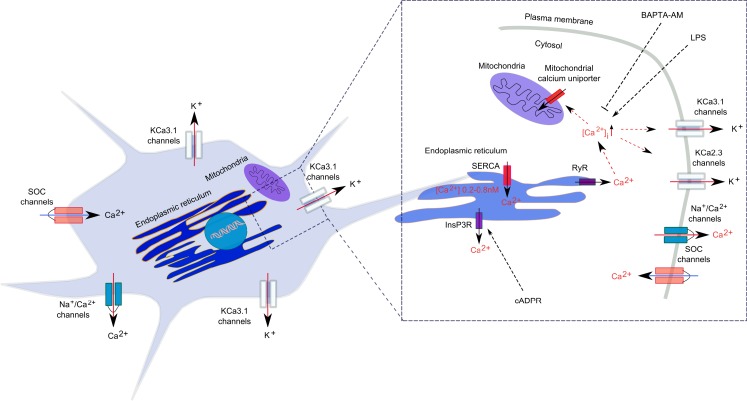
**Calcium regulation in microglia**. Calcium signal generation is achieved by a well-regulated relationship between Ca^2+^ release from the intracellular stores and the Ca^2+^ entry through plasmalemma. In the ER, sarcoendoplasmic reticulum Ca^2+^-ATPases (SERCA) transfer Ca^2+^ to the lumen of the ER, while the Ca^2+^ release from ER into the cytoplasm is accomplished by ryanodine receptors (RyRs) and inositol 1,4,5-triphosphate (IP_3_)-gated calcium channels. Ca^2+^ also accumulates in mitochondria through a Ca^2+^-selective uniporter. Ca^2+^ extrusion from the cytosol is achieved by a Na^+^/Ca^2+^ exchanger.

In microglial cells, intracellular calcium signals are modulated by calcium diffusion through membrane ion channels and by active and passive transport through calcium pumps and co-transporters (Kettenmann et al., 2011; Figure 1). Like in all non-excitable cells, Ca^2+^ signals in microglia are regulated by Ca^2+^ release mechanisms from the intracellular stores and by extracellular Ca^2+^ entry into the cytosol through membrane-located store-operated Ca^2+^ (SOC) channels and ligand-gated channels (Kettenmann et al., 2011). Release of free Ca^2+^ into the cytosol is mainly attributed to the dynamic release from intracellular stores, such as ER and mitochondria. In the ER, sarcoendoplasmic reticulum Ca^2+^-ATPases (SERCA) transfer Ca^2+^ to the lumen of the ER, while the Ca^2+^ release from ER into the cytoplasm is accomplished by RyRs and IP_3_-gated calcium channels (Verkhratsky and Kettenmann, 1996; Burdakov et al., 2005; Klegeris et al., 2007).

Although there is no evidence so far for an association between SK/IK/K_Ca_2/K_Ca_3 channels and RyR receptors in microglia, it was demonstrated in neurons of the rat and mouse substantia nigra pars reticulata and the rat medial preoptic nucleus, that RyR-mediated Ca^2+^ release from intracellular stores activated SK2/K_Ca_2.2 and SK3/K_Ca_2.3 channels, respectively (Yanovsky et al., 2005; Klement et al., 2010). Further, combined electrophysiological, immunohistochemical, and two-photon Ca^2+^ imaging techniques applied to the rat nucleus reticularis thalami indicated that calcium-induced calcium release (CICR) via RyRs activated plasma membrane SK2/K_Ca_2.2 channels, which together with SERCA pumps and low-voltage-activated Ca^2+^ channels, shaped rhythmic [Ca^2+^]_i_ oscillations (Coulon et al., 2009). In rat smooth muscle cells, CICR have a critical implication in the regeneration of the contractile cycle, since Ca^2+^ release via RyRs facilitates the activation of IK/K_Ca_3.1 channels, which, in turn, mediates smooth muscle cell hyperpolarization and relaxation (Haddock and Hill, 2002). Since both RyR receptors and SK/IK/K_Ca_2/K_Ca_3.1 channels are expressed and functional in microglial cells, research on expression, and function of K_Ca_ channels in the ER requires further in depth investigation in order to demonstrate their functional interconnectivity, potential role in the regulation of intracellular calcium homeostasis, and influence on inflammatory responses in activated microglia.

Several studies have reported that inflammatory activation promoted dysbalanced calcium homeostasis in microglia (Hoffmann et al., 2003; Beck et al., 2008; Kettenmann et al., 2011). For example, LPS triggered mouse primary microglial activation, NO, and cytokine release, an increase in [Ca^2+^]_i_, and a decrease of calcium signals in response to UTP and complement factor 5a (Hoffmann et al., 2003). The critical role of [Ca^2+^]_i_ in microglial activation was demonstrated by the intracellular calcium chelator BAPTA-AM that reverted LPS-induced microglial activation and reduced the associated NO and cytokine production in both mouse and rat primary microglia (Hoffmann et al., 2003; Nagano et al., 2006). Extracellular Ca^2+^ is likely of major importance for microglial activation, since depletion of extracellular Ca^2+^ or EDTA diminished LPS-induced microglial activation and proliferation *in vitro* in mouse primary microglia (Dolga et al., 2012). Interestingly, an increase in [Ca^2+^]_i_ is more a facilitator than a trigger of microglial activation, since, for example, ionomycin increased [Ca^2+^]_i_ but it did not induce cytokine or NO releases from microglia (Hoffmann et al., 2003). Studies addressing the influence of calcium homeostasis on cell survival pathways demonstrated that extracellular calcium chelation did not trigger microglial cell death, whereas increasing [Ca^2+^]_i_ with ionomycin or thapsigargin induced apoptotic cell death (Hoffmann et al., 2003; Nagano et al., 2006). Furthermore, in LPS-stimulated microglia, thapsigargin and ionomycin induced necrotic cell death, and these effects were attenuated by lowering [Ca^2+^]_i_ with BAPTA-AM (Nagano et al., 2006). These data suggest that deregulated [Ca^2+^]_i_ concentration in activated microglia is critical for cell survival and shifts the mode of cell death from apoptosis to necrosis (Hoffmann et al., 2003; Nagano et al., 2006). Better understanding of the consequences of deregulated intracellular Ca^2+^ concentration in microglial cells warrants comprehensive investigation for establishing potential therapeutic approaches for inflammation-related CNS disorders.

## Protective Role of SK/K_ca_2 Channels in Neurons and Microglia

Several acute and chronic pathologies, including Alzheimer and Parkinson’s disease, multiple sclerosis, ALS, cerebral ischemia, and cardiovascular disease are endowed with an inflammatory component. The function of SK/K_Ca_2 channel in stroke and Alzheimer’s disease was recently reviewed by Kuiper et al. (2012) and on atherosclerosis and cardiovascular diseases by Wulff and colleagues (Wulff et al., 2007; Köhler et al., 2010). Neurodegenerative diseases triggered by neuronal hyperexcitability, progressive dysregulated Ca^2+^ homeostasis, and excitotoxic neuronal death could benefit from small molecules that enhance SK/K_Ca_2 channel activity (Kuiper et al., 2012). Other reviews present data on opening of endothelial K_Ca_3.1/K_Ca_2.3 channels, which stimulate endothelium-derived-hyperpolarizing-factor (EDHF)-mediated arteriolar dilation (Brähler et al., 2009) and lower blood pressure (Wulff et al., 2007; Köhler et al., 2010). On the other hand, inhibition of smooth muscle IK/K_Ca_3.1 channels is considered for the treatment of pathological vascular remodeling and sickle cell anemia, since IK/K_Ca_3.1 channel inhibition has particular beneficial effects in restenosis disease, atherosclerosis, and autoimmune encephalomyelitis (Wulff et al., 2007; Köhler et al., 2010).

Further, potassium IK1/SK4/K_Ca_3.1 channels are also under intense investigation because of their high potential in therapeutic drug development for various pathophysiological conditions, including sickle cell anemia, pancreatic cancer, arterial restenosis, immune diseases, and CNS inflammation (Jensen et al., 2002; Köhler et al., 2003; Wulff et al., 2003; Jager et al., 2004). In particular, IK/K_Ca_3.1 channels are highly expressed in murine microglial cells (Khanna et al., 2001) and are implicated in the production of reactive oxygen species, nitric oxide as well as peroxynitrite and protein tyrosine nitration (Skaper, 2011). Increasing evidence for beneficial effects of IK/K_Ca_3.1 channel inhibition is mainly derived from studies using the selective antagonist triarylmethane-34 (TRAM-34), which does not exert any action on *KCNN1*-3/SK1-3/K_Ca_2.1-K_Ca_2.3 channels (Wulff et al., 2000). Interestingly, TRAM-34 reduces the neurotoxicity mediated by LPS-activated rat primary microglia, and this effect is associated with diminished iNOS expression and reduced caspase 3 activation (Kaushal et al., 2007). TRAM-34 also reduced the degeneration of retinal ganglion cells after optic nerve transection in adult female Sprague Dawley rats (Kaushal et al., 2007). It improved locomotor function, reduced the secretion of TNF-α and interleukin-1β, and diminished the expression of iNOS in a rodent model of spinal cord injury (Bouhy et al., 2011). In a rat model of ischemia/reperfusion stroke, TRAM-34 reduced infarct area by ≈50% as determined by hematoxylin and eosin staining and improved neurological deficits related to the secondary inflammatory damage (Chen et al., 2011).

Neuroinflammation and dysfunction of dopaminergic midbrain neurons have been implicated in the etiology of Parkinson’s disease (Qian et al., 2010; Whitton, 2010). Low-level activity of dopaminergic neurons and alterations of dopamine release have been associated with impairment in voluntary movements, working memory, and reward-based learning (Dunnett and Bjorklund, 1999; Spanagel and Weiss, 1999; Svensson, 2000). Therefore, it is of paramount importance to identify molecular mechanisms underlying dopaminergic activity and neurotransmitter release. During early postnatal development, dopaminergic neurons display anomalous firing patterns and exhibit spontaneous miniature hyperpolarizations, which are not present in adults (Cui et al., 2004). These spontaneous hyperpolarizations exhibit outward currents and depend on SK/K_Ca_2 channel regulation, T-type Ca^2+^ channel activation, and RyR-dependent Ca^2+^-induced Ca^2+^ release in mouse and rat midbrain areas (Wolfart et al., 2001; Cui et al., 2004). *In vivo* recordings demonstrated that adult rat dopaminergic neurons exhibit either a single-spike pacemaker in a burst firing pattern or show irregular firing patterns (Wilson et al., 1977; Grace and Bunney, 1984), while *in vitro* recordings in rat dopamine neurons demonstrated a regular, low-frequency pacemaker activity (Grace and Onn, 1989). These discrepancies between *in vitro* and *in vivo* recordings reside in a particular type of synaptic activity able to switch dopaminergic neurons into burst firing (Cui et al., 2004).

SK/K_Ca_2 channels are emerging candidates to control NMDAR activation (Ping and Shepard, 1996), GABA receptor-mediating inhibitory activities (Yanovsky et al., 2005) and postsynaptic conductances in dopaminergic neurons. In fact, a subtype of SK/K_Ca_2 channels, SK3/K_Ca_2.3 subtype was shown to be expressed in mouse, rat, and guinea pig dopaminergic neurons (Wolfart et al., 2001; Bosch et al., 2002; Sarpal et al., 2004; Benítez et al., 2011). The function of SK3/K_Ca_2.3 channels was addressed in mouse dopaminergic neurons of the substantia nigra, where they regulate the frequency and precision of pacemaker spiking (Wolfart et al., 2001). However, SK3/K_Ca_2.3 channels were not found in a subpopulation of mouse dopaminergic neurons in the ventral tegmental area (Wolfart et al., 2001), suggesting that these significant differences in the density of apamin-binding sites in dopaminergic neurons might be responsible for the functional differences between ventral tegmental area (A10) and substantia nigra (A9; Grenhoff et al., 1988). Recently, immune-electron microscopy established also the presence of SK2/K_Ca_2.2 channel subtype in mouse dopaminergic neurons (Deignan et al., 2012). SK2/K_Ca_2.2 channels are expressed exclusively in the dendrites of dopaminergic neurons, while SK3/K_Ca_2.3 channels are expressed in the soma and, to a lesser extent, in the dendritic neuronal network. To gain further knowledge of the precise function of SK/K_Ca_2 channels in dopaminergic neurons, experiments performed in subtype specific null mice have demonstrated that SK2/K_Ca_2.2-containing channels are responsible for the precision of action potential timing, while SK3/K_Ca_2.3-containing channels contribute to action potential frequencies (Deignan et al., 2012). Indeed, modulation of SK/K_Ca_2 channels was shown to regulate the excitability of rat midbrain neurons (Ji et al., 2009). Positive SK/K_Ca_2 channel modulation using a non-specific SK/K_Ca_2 channel subtype compound, NS309 (Strøbaek et al., 2004) decreased the responsiveness of rat dopaminergic neurons to depolarizing currents, enhanced spike frequency adaptation, and slowed spontaneous firing, due to an increase in the amplitude and duration of AHP (Ji et al., 2009).

Further, CyPPA, a positive modulator of SK2/K_Ca_2.2 and SK3/K_Ca_2.3 channel subtypes (Hougaard et al., 2007), decreases spontaneous firing rate and increases the duration of the apamin-sensitive AHP, causing an activity-dependent inhibition of current-evoked action potentials in both mouse and rat midbrain slices via SK3/K_Ca_2.3 channel activation (Herrik et al., 2012). Although CyPPA repressed dopamine release in a concentration-dependent manner *in vivo* systemic administration of CyPPA attenuated methylphenidate-induced hyperactivity and stereotypic behaviors in mice (Herrik et al., 2012). *In vitro* studies further demonstrated that CyPPA prevented neuronal loss in an AMPA-dependent toxicity model in rat dopaminergic neurons (Benítez et al., 2011). In contrast, negative pharmacological modulation by NS8593 (Strøbaek et al., 2006), a non-specific SK/K_Ca_2 channel subtype compound increased rat dopaminergic excitability, induced an irregular pacemaker and bursting discharge (Ji et al., 2009) and reduced the number of rat dopaminergic neurons in a dose-dependent manner (Benítez et al., 2011).

Taken together, modulation of SK/K_Ca_2 channels in dopaminergic neurons regulates neuronal excitability, survival, and neurotransmitter release, making them suitable candidates for therapeutic intervention in pathological conditions related to dopaminergic dysfunction, such as Parkinson’s disease.

Disturbed Ca^2+^ homeostasis is one of the major causes of delayed cell death and infarct development after acute brain damage, e.g., after cerebral ischemia. Consequently, reducing intracellular [Ca^2+^]_i_ levels by blocking NMDA receptors has been widely investigated in experimental and clinical stroke studies. However, inhibitors of NMDA receptors largely failed in clinical studies and, therefore, novel strategies controlling [Ca^2+^]_i_ homeostasis are warranted for the development of effective therapies. An alternative approach avoiding direct NMDA receptor targeting would be to modulate [Ca^2+^]_i_ homeostasis indirectly via [Ca^2+^]_i_ sensors, such as K_Ca_ channels. Earlier studies showed that pyramidal cells from the CA3 area of the rat hippocampus respond to chemical-induced ischemia with an initial transient hyperpolarization that is responsible for delayed neuroprotective effects (Tanabe et al., 1999). A potential neuroprotective role for SK/K_Ca_2 channels was identified when apamin-sensitive SK/K_Ca_2 conductance channels were activated and generated a pronounced outward current in response to brief ischemia in rat hippocampal organotypic slice cultures (Tanabe et al., 1999). Indeed, activation of SK2/K_Ca_2.2 channels by NS309 or 1-EBIO positive modulators decreased brain damage area (Allen et al., 2011; Dolga et al., 2011) and also regulated calcium homeostasis (Dolga et al., 2011). In addition, SK2/K_Ca_2.2 channel activation is required for neuroprotection in neurons against glutamate-induced excitotoxicity and also *in vivo* in a mouse model of middle cerebral artery occlusion (MCAo; Allen et al., 2011; Dolga et al., 2011).

## Summary and Perspectives

Neuroinflammation is a critical component for the initiation and progression of several neurodegenerative pathologies, such as cerebral ischemia, schizophrenia, Parkinson’s, and Alzheimer’s diseases. Potential therapeutic approaches directed against both inflammation and neuronal degeneration might benefit from modulation of SK/IK/K_Ca_ channels since SK/K_Ca_2 channel activity elicits a dual mechanism of action with direct protective effects in neuronal cells and inhibition of inflammatory activities in microglial cells. However, the role of SK/IK/K_Ca_ channels in microglial activity and maintenance needs further in depth investigation in order to reveal the culpable molecular mechanisms. It is of interest to determine whether SK/IK/K_Ca_ channels are endowed with similar characteristics among different species, whether their activity alters during aging or during the progression of neurodegeneration, and whether modulating SK/IK/K_Ca_ channel activity can limit neurodegeneration and related neuroinflammation. Altogether, the hitherto accumulated evidence exposes modulation of SK/IK/K_Ca_ channel functions as potential target in neurodegenerative diseases where inflammatory processes significantly contribute to the progress of neuronal dysfunction.

## Conflict of Interest Statement

The authors declare that the research was conducted in the absence of any commercial or financial relationships that could be construed as a potential conflict of interest.
